# The Institutional Worker

**Published:** 1912-03-16

**Authors:** 


### INSTITUTIONAL WORKER

Being a Special Supplement to "The Hospital"

### Editor's Notices.

**Contributions:**

Contributions should be written, or preferably typed, on one side of the paper only, and all articles sent in are accepted upon the distinct understanding that they are forwarded to THE HOSPITAL only.

The Editor cannot undertake to return MSS. not used, but when a stamped directed envelope is enclosed the MSS. may be returned if a special request is made.

Accepted articles and paragraphs of news will be paid for after publication at the scale rate.

**Address:**

To prevent delay all contributions and letters on editorial business must be addressed exclusively to the Editor, "The Hospital," 29 Southampton Street, Strand, London, W.C. It is important that this regulation shall be strictly observed.

**Correspondence:**

Correspondence on all subjects is invited. The name and address of correspondents must be given as a guarantee of good faith, but not necessarily for publication.

**Special Articles:**

Special articles are invited, and questions, inquiries, and paragraphs upon all matters relating to the work, administration, and management of general, special, mental, fever, cottage and convalescent hospitals, sanatoria, homes, institutions, societies and organisations for the treatment or care of the sick, injured and dependents of all classes. Every matter affecting the interests and welfare of the staff of all grades working in these institutions will receive special consideration.

**Administrative Medicine, Research and Items of News:**

Special terms will be paid for approved articles on subjects in this wide field by experts who have made some section of it their special study and interest. We wish to give prominence to every movement tending to advance accuracy of diagnosis, efficiency in treatment, and the development of modern methods to eradicate disease and promote the welfare of the suffering.

**A Bureau of Information:**

We invite inquiries and applications for information and help in providing for that numerous class of sufferers who require special care or house-room which they cannot obtain in their own homes or secure for themselves. We cannot, however, prescribe or recommend practitioners.

**Books for Review:**

Publishers are particularly requested to send advance proofs of any new books of importance whenever possible, as the Editor has made arrangements to publish immediate reviews on a new plan.

**Photographs, Plans, Blocks, and Illustrations:**

It is requested that wherever possible MSS. may be accompanied by illustrations in any of the above forms. The name of the sender and of the article to which it belongs should be written on the back of each photograph, drawing, or block for purposes of identification.

**Local Papers:**

Newspapers containing reports or news paragraphs should be marked and addressed to the Sub-Editor.

**The Coupon System:**

A coupon will be found at the bottom of the third inside page of the cover each week. This coupon must be attached to every question or inquiry to which an answer is desired in THE HOSPITAL.

### Institutional Authorities and "The Institutional Worker."

**Staff Appointments:**

AN intelligent and capable staff is absolutely essential to the efficient Administration and Management of any Institution, and in order to fill such vacancies as occur from time to time with the best available material, it is necessary to secure the widest possible publicity among those trained to and experienced in institutional work. As the Journal of the Institutional Worker, THE HOSPITAL is quite without comparison as an effective medium for all notices of Appointments Vacant, no matter in what department of Institutional Life, and authorities will find it to their advantage to avail themselves of the SPECIAL RATES which are allowed to those institutions that agree for one year to send all their public notices for insertion in this Section of THE HOSPITAL.

**Supplies:**

THE economical management of an Institution depends very largely upon the purchase of supplies in the cheapest market. And as it is often impossible locally to secure the highest quality at the lowest prices, a column is reserved in this part of the paper for announcements of Institutions inviting Tenders for Contracts.

This enables Authorities of Institutions to secure tenders for supplies from all over the country, and ensure purchases at the lowest possible prices consistent with quality.

The charge for Tenders is 6s. per inch, and for this small outlay many pounds may be saved on supplies of all descriptions.

### Manager's Notices.

**Letters relating to the publication, sale, and advertising departments of "The Hospital," must be addressed to The Manager, "The Hospital," The Hospital Building, 28 and 29 Southampton Street, London, W.C.**

**Subscriptions** may begin at any time, and are payable in advance. Cheques and Post-Office orders should be crossed London County and Westminster Bank, Covent Garden branch, and made payable to the Manager of THE HOSPITAL.

RATES OF SUBSCRIPTION (payable in advance).

| | |
|---|---|
| UNITED KINGDOM. | |
| Three Months (including Postage) | 2s. 0d. |
| Six Months do. | 4s. 0d. |
| Twelve Months do. | 6s. 6d. |
| FOREIGN AND COLONIAL. | |
| Three Months (including Postage) | 2s. 6d. |
| Six Months do. | 5s. 0d. |
| Twelve Months do. | 8s. 8d. |

**Advertisements:**

To ensure insertion, all advertisements for THE HOSPITAL must reach the Manager not later than Wednesday morning in each week. For scale of charges see pages vii and 2.

**Remittances:**

**It is especially requested that remittances be made by cheque or Postal Order, and in halfpenny stamps only when the amount is under sixpence.**

### OFFICIAL APPOINTMENTS VACANT.

### Poor-Law Vacancies.

Assistant Attendant (£22), Brentford Union. Matron, The Infirmary, Isleworth, W.

Assistant Clerk (£70), Bermondsey Guardians. Mr. E. Pitts Fenton, C. to G., 283 Tooley Street, S.E. March 18.

Assistant Cook (£24), Bermondsey Guardians. The C. to G., 283 Tooley Street, S.E. March 18.

Assistant Labour Master (£30), Wandsworth Union. Mr. F. W. Piper, C. to G., St. John's Hill, Wandsworth, S.W. March 27.

Assistant Matron (£40), Wandsworth Union. Mr. F. W. Piper, C. to G., St. John's Hill, Wandsworth, S.W, March 27.

Assistant Nurse (£25), Peterborough Union. Mr. I. Whitsed, C. to G., Peterborough. March 16.

Assistant Nurse (£27 10s.), Alverstoke Guardians. Mr. F. B. Bulmer, C. to G., Gosport. March 18.

Assistant Nurse (£26), Bucklow Union. The C. to G., Knutsford. March 18.

Assistant Nurse (£22), Bosmere and Claydon Union. Mr. R. M. Cook, C. to G., 6 Providence Street, Ipswich. March 19.

Assistant Nurse (£25), Hereford Union. Mr. R. Moore, C. to G., Hereford. March 21.

Assistant Nurse (£28), Bromley Union. G. Haslehurst, C. to G., Union Offices, Park House, Bromley, Kent. March 25.

Assistant Nurse (£22), Bermondsey Guardians. Matron of the Schools, Wickham Road, Shirley, Croydon.

Assistant Nurse (£25), East Retford Union. J. G. Dimock, C. to G., Retford.

Assistant Nurse (£28), Newton Abbot Union. F. Horner, C. to G., Union Offices, Newton Abbot. March 19.

Assistant Nurse (£20), Falmouth Union. R. N. Rogers, C. to G., Union Offices, Falmouth. March 26.

Assistant Nurses (2) (£18), Holborn Union. Matron of the Workhouse, Mitcham, Surrey.

Assistant Relieving Officer (£120), Islington Guardians. Mr. E. Davey, C. to G., St. John's Road, Upper Holloway, N. March 18.

Boys' Attendant and Tailor (£24), Tiverton Union. Mr. J. F. Pugsley, C. to G., Tiverton. March 18.

Case-Paper Clerk (£65), Burton-upon-Trent Union. Mr. C. F. Chamberlin, C. to G., Burton-on-Trent. March 23.

Case-Paper Clerks (3) (30s., 15s., and 10s. weekly), Cardiff Union. Mr. A. J. Harris, C. to G., Cardiff. March 19.

Charge Nurse (£35), Hartismere Union. H. Warnes, C. to G., Union Offices, Eye, Suffolk. March 22.

Charge Nurse (£30), Isle of Wight Union. Mr. A. G. Harrison, C. to G., Newport, I.W. March 20.

Charge Nurse (£30), Scarborough Union. J. W. Read, C. to G., Scarborough. March 20.

Charge Nurse (£35), Bromley Union. E. Haslehurst, C. to G., Union Offices, Park House, Bromley, Kent. March 18.

Charge Nurse (£32), Walsall Union. A. H. Lewis, C. to G., 29 Leicester Street, Walsall. March 14.

Charge Nurse (£30), Oldham Union. H. A. Quarmby, C. to G., Rochdale Road, Oldham. March 12.

Charge Nurse (£32), Bedford Union. W. Payne, C. to G., 115 High Street, Bedford. March 30.

Charge Nurses (£30), Rochford Union. Mr. F. Gregson, C. to G., Southend-on-Sea. March 18.

Children's Attendant (£20), Cockermouth Union. J. H. Musgrave, C. to G., Grecian Villa, Cockermouth.

Children's Attendant (£17), Farnham Union. Miss Tisdell, Superintendent Nurse, The Infirmary, Farnham.

Cook (£22), Lutterworth Union. Mr. T. C. Bodycote, C. to G., Lutterworth. March 20.

Female Attendant (£25), Devizes Union. Mrs. Fear, Matron of the Workhouse, Devizes.

Female Cook (£40), Poplar Guardians. Mr. G. H. Lough, C. to G,. Upper North Street, Poplar. March 18.

Female Imbecile Attendant (£25), Keighley Union. Mr. S. Green, C. to G., Keighley. March 18.

Female Imbecile Attendant (£30), Burnley Union. Mr. J. S. Horn, C. to G., Burnley. March 13.

Foster-Mother (£25), Bucklow Union. The C. to G., Knutsford. March 18.

Foster-Mother (£22), Newton Abbot Union. Mr. F. Horner, C. to G., Newton Abbot. March 19.

Foster-Mother (£25), Ecclesall Bierlow Union. Mr. J. E. Moulding, C. to G., The Edge, Sheffield. March 16.

Foster-Mother (£25), Keighley Union. Mr. S. Green, C. to G., Keighley. March 18.

General Female Assistant (£20), Barton-on-Irwell Union. Mr. J. W. Whitworth, C. to G., Patricroft. March 19.

Headmaster's Clerk and Storekeeper (£30), Islington Guardians. Headmaster of the Schools, Hornsey, N.

Head Sister and Superintendent Nurse (£35), Ashton-under-Lyne Union. G. H. Partington, C. to G., Poor Law Offices, Ashton-under-Lyne. March 26.

Hospital Cook (Female) (£27), Burton-upon-Trent Union. Mr. C. F. Chamberlin, C. to G., Burton-on-Trent. March 23.

Industrial Trainer (Dressmaker) (£26), Portsmouth Guardians. Mr. E. H. Mitchell, C. to G., Portsmouth. March 25.

Labour Master (£30), Fulham Guardians. Mr. E. J. Mott, C. to G., 129 Fulham Palace Road, W. March 18.

Labour Mistress, etc. (£25), Lewisham Union. Mr. H. C. Mott, C. to G., 394 High Street, Lewisham, S.E. March 16.

Laundress (£30), Henley Union. Mr. A. R. Lloyds, C. to G., Henley-on-Thames.

Laundress (£22 10s.), Chesterfield Union. Mr. R. F. Hartwright, C. to G., Chesterfield. March 16.

Laundress (£25), Kidderminster Union. Mr. F. E. Burcher, C. to G., Kidderminster. March 18.

Male Cook (£35), Horsham Union). Mr. A. C. Coole, C. to G., Horsham. March 19.

Male Relief Attendant (£35), Bethnal Green Guardians. Master of the Workhouse, Waterloo Road, N.E.

Master and Matron (£200 and £90), West Ham Union. Mr. T. Smith, C. to G., Leytonstone, N.E. March 28.

Master and Matron (£50 and £37 10s.), Chard Union. Mr. F. G. Ross, C. to G., Chard. March 16.

Maternity Sister (£35), Oldham Union. H. A. Quarmby, C. to G., Rochdale Road, Oldham. March 12.

Needlewoman (£22), Hackney Union. Matron of the Children's Homes, Ongar, Essex.

Nurse (£35), Lanchester Union. W. H. Ritson, C. to G., Lanchester, Durham. March 27.

Nurse (£28), West Ashford Union. Mr. J. M. Poncia, C. to G., Ashford, Kent. March 18.

Nurse (£35), Pontardawe Union. Mr. W. Lewis, C. to G., Pontardawe, Glam. March 18.

Nurse (£30), Chester-le-Street Union. Mr. R. V. Dickinson, C. to G., Chester-le-Street. March 18.

Nurse (£65, non-resident), Holywell Union. Mr. P. H. Roberts, C. to G., Holywell. March 22.

Nurse (£30), Stockbridge Union. Matron of the Workhouse, Stockbridge.

Nurse (£26), Paddington Guardians. H. F. Aveling, C. to G., 313 Harrow Road, W. March 20.

Nurse (£26), Langport Union. E. Q. Louch, C. to G., Bank Chambers, Langport, Som. March 18.

Nurse (£30), Ormskirk Union. A. Dickinson, C. to G., Ormskirk. March 13.

Nurses (£26), Doncaster Union. Mr. H. M. Marshall, C. to G., Doncaster.

Nurses (£25), Richmond Union. P. Umney, C. to G., Guardians' Offices, Parkshot, Richmond, Surrey. April 12.

Porter, etc. (£25), Biggleswade Union. Mr. G. Wagg, C. to G., Biggleswade. March 16.

Probationer (£15), Braintree Union. A. Hills, C. to G., Union Offices, The Workhouse, Bocking, Essex. March 25.

Probationer Nurse (£12), Kettering Union. C. W. Lane, C. to G., George Street, Kettering. March 21.

Probationer Nurses (£12), St. George's Union. Matron, St. George's Infirmary, Fulham Road, S.W.

